# *Porphyromonas gingivalis*-induced periodontitis could contribute to cognitive impairment in Sprague–Dawley rats via the P38 MAPK signaling pathway

**DOI:** 10.3389/fncel.2023.1141339

**Published:** 2023-03-28

**Authors:** Ru Jin, Xiaoqiao Ning, Xiang Liu, Yueyang Zhao, Guo Ye

**Affiliations:** ^1^Chongqing Key Laboratory of Oral Disease and Biomedical Sciences and Chongqing Municipal Key Laboratory of Oral Biomedical Engineering of Higher Education, Stomatological Hospital, Chongqing Medical University, Chongqing, China; ^2^The First People’s Hospital of Wanzhou, Chongqing, China; ^3^Department of Anatomy, Chongqing Medical University, Chongqing, China

**Keywords:** cognitive impairment, periodontitis, neuroinflammation, APP processing, P38 MAPK signaling pathway

## Abstract

**Background:**

Periodontitis is one of the most common oral diseases and has been shown to be a risk factor for systemic diseases. Our aim was to investigate the relationship between periodontitis and cognitive impairment and to explore the role of the P38 MAPK signaling pathway in this process.

**Methods:**

We established a periodontitis model by ligating the first molars of SD rats with silk thread and injecting *Porphyromonas gingivalis* (*P. gingivalis*) or *P. gingivalis* plus the P38 MAPK inhibitor SB203580 at the same time for ten weeks. We assessed alveolar bone resorption and spatial learning and memory using microcomputed tomography and the Morris water maze test, respectively. We used transcriptome sequencing to explore the genetic differences between the groups. The gingival tissue, peripheral blood and hippocampal tissue were assessed for the cytokines TNF-α, IL-1β, IL-6, IL-8 and C reactive protein (CRP) with enzyme-linked immunosorbent assay (ELISA) and reverse transcription polymerase chain reaction (RT–PCR). We observed the presence of *P. gingivalis* in the hippocampus of rats by paraffin-fluorescence in situ hybridization (FISH). We determined the activation of microglia by immunofluorescence. Finally, Western blot analysis was employed to determine the expression of amyloid precursor protein (APP), β-site APP-cleaving enzyme 1 (BACE1) and P38MAPK pathway activation.

**Results:**

We demonstrated that silk ligature-induced periodontitis plus injection of *P. gingivalis* into subgingival tissue could lead to memory and cognitive impairment. Transcriptome sequencing results suggested that there were neurodegenerative diseases in the *P. gingivalis* group, and the MWM test showed that periodontitis reduced the spatial learning and memory ability of mild cognitive impairment (MCI) model rats. We found high levels of inflammatory factors (TNF-α, IL-1β, IL-6, and IL-8) and CRP in the gingiva, peripheral blood and hippocampus, and the expression of APP and BACE1 was upregulated, as was the P38 MAPK pathway activation. Activated microglia and the presence of *P. gingivalis* were also found in the hippocampus. P38 MAPK inhibitors mitigated all of these changes.

**Conclusion:**

Our findings strongly suggest that topical application of *P. gingivalis* increases the inflammatory burden in the peripheral and central nervous systems (CNS) and that neuroinflammation induced by activation of P38 MAPK leads to impaired learning and memory in SD rats. It can also modulate APP processing. Therefore, P38 MAPK may serve as a linking pathway between periodontitis and cognitive impairment.

## Introduction

Periodontitis is a dysbiotic inflammatory disease induced by bacteria, which often causes severe damage to the periodontal support tissue and even leads to tooth loss (Eke et al., 2020; Daghrery et al., 2023). Globally, the prevalence of the disease is as high as 11.2%, affecting 743 million individuals, making it the sixth most common human disease (Richards, 2014; Sanz et al., 2020; Kwon et al., 2021). Human subgingival plaque biofilms contain more than 500 species of bacteria. Under the synergistic effect of other commensal bacteria, *Porphyromonas gingivalis* (*P. gingivalis*), which is one of the major pathogenic bacteria causing periodontal disease, can further trigger alveolar bone resorption (Hajishengallis, 2015; Zhang et al., 2020). This “red-complex” perio-pathogen produces numerous virulence factors that directly or indirectly damage periodontal tissue by modulating the host inflammatory response (How et al., 2016). The host inflammatory immune response produces factors that cause inflammation, it provides nutrients for the survival of periodontal pathogenic bacteria dominated by *P. gingivalis*, which further causes damage to periodontal tissues (Brunner et al., 2010). In addition to causing damage to periodontal tissues, these inflammatory factors can also have certain effects on systemic organs, such as distant brain. Numerous studies have shown that periodontitis is closely related to systemic diseases, including hypertension, cardiovascular disease, diabetes, and mild cognitive impairment (MCI) (Preshaw et al., 2012; Hamilton et al., 2017; Larvin et al., 2022).

Mild cognitive impairment can be considered a precursor to the neurodegenerative disease dementia and is pathologically characterized by senile plaques and neurofibrillary tangles. Amyloid precursor protein (APP) and activation of β-site APP-cleaving enzyme 1 (BACE1) are specific pathological markers (Hardy and Higgins, 1992; Winblad et al., 2004; Dunne et al., 2021). A study showed that the disease has become the sixth leading cause of death and ranks fifth in the fatality rate in the over-65-year-old age group (Alzheimer’s Association Report, 2021). Inflammation is thought to play a significant role in MCI. The inflammatory responses can originate from periodontitis, an inflammatory process associated with periodontal colonization bacteria (Dioguardi et al., 2020). Clinical evidence has demonstrated a link between periodontitis and MCI (Noble et al., 2009; Ide et al., 2016; Nilsson et al., 2018). *P. gingivalis* is one of the main pathogens related to periodontal disease, and it is closely related to many systemic diseases, including MCI (Poole et al., 2013; Zhang et al., 2018; Díaz-Zúñiga et al., 2020). *P. gingivalis* can cause a local periodontal inflammatory response and produce inflammatory mediators, such as IL-1β, IL-8, and TNF-α. With the continuous progression of periodontal inflammation, proinflammatory cytokines can lead to systemic inflammation and may reach the central nervous system through systemic circulation (Watts et al., 2008). In addition, periodontal bacteria and their active ingredient can invade the systemic circulation of patients with periodontal disease (Bretz et al., 2005). Inflammatory mediators produced by periodontal pathogens and active components of bacteria can activate microglia in the brain to trigger neuroinflammation and ultimately lead to MCI (Wu and Nakanishi, 2014).

P38 MAPK is highly expressed in regions of the central nervous system that are critical for learning and memory (Yasuda et al., 2011). Indeed, the activation of P38 MAPK has been verified in the brain during the MCI, both clinically and in mouse models (Pei et al., 2001; Kheiri et al., 2018). In a previous study, Sun et al. observed the P38 MAPK pathway in the postmortem brains of neurodegenerative disease patients and animal models (Sun et al., 2003; Schnöder et al., 2016). Recently, according to cross-linking in four datasets, researchers found genetic crosstalk that links periodontitis and Alzheimer’s disease (AD), and crosstalk genes were found to be regulated by several pathways, including JAK-STAT, MAPK, and NF-κB (Jin et al., 2021). Some researchers have confirmed that MCI is associated with neuroinflammation and that the TLRS/NF-κB and STAT3 pathways all play a significant role in the association between MCI and periodontitis (Hu et al., 2020, 2021). However, whether P38 MAPK is present in periodontitis-induced MCI has not yet been fully clarified.

In this study, the Morris water maze (MWM) test was employed to observe the effect of ligation of the maxillary first molar plus subgingival tissue injection on periodontitis development and the learning and memory of rats. We employed transcriptome sequencing to elucidate genetic differences between groups. The activation of microglia and the existence of *P. gingivalis* in the brain were observed by immunofluorescence and fluorescence probe-FISH. Inflammatory factors were detected in the brain using ELISA and RT–PCR, as well as the detection of inflammatory factors in the gingiva and peripheral blood with ELISA. In addition, APP processing and P38 MAPK signaling pathway activation were assessed by Western blotting.

## Materials and methods

### Animals and treatment

Twenty-four 12-week-old male SD rats were purchased from Chongqing Medical University. All rats were housed at a standard temperature of 20 ± 2°C and fed food and water under a 12-h light/dark cycle. Feeding was according to the National Research Council’s Guide for the Care and Use of Laboratory Animals. *In vivo* experiments of rats, we fully comply with ARRIVE guidelines. This project has been approved by the Ethics Committee of the Stomatological Hospital of Chongqing Medical University, Chongqing, China (No. 067, 2022).

**FIGURE 1:**
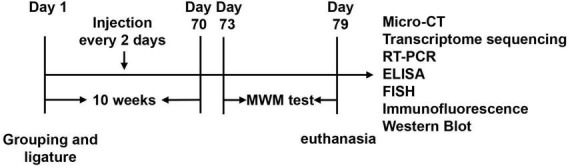
Experimental design. After ten weeks of injection, we underwent a six-day MWM test. The underlying mechanism was further detected by Micro-CT, transcriptome sequencing, RT-PCR, ELISA, Paraffin-Fluorescence *in situ* hybridization (FISH), Immunofluorescence, and Western blot.

Twenty-four SD rats were randomly divided into three groups: the control group (control), the *Porphyromonas gingivalis* group (*P. gingivalis*), and the *Porphyromonas gingivalis* + SB203580 group (*P. gingivalis* + SB). SB203580 is a P38MARK pathway inhibitor that can efficiently inhibit kinase activity in the signaling pathway (Davies et al., 2000). We induced experimental periodontitis using 3-0 silk ties in the cervical subgingival region of the maxillary first molars in the *P. gingivalis* and *P. gingivalis* + SB groups after they were gas-anesthetized with isoflurane (maintain anesthesia concentration: 2.5%). The *P. gingivalis* group was then injected with 3 μl of *P. gingivalis* (1 × 10^9^ CFU/ml) into the subgingival tissue of the maxillary first molars with a 5-μl microsyringe (Hamilton, Switzerland) every 2 days for 10 weeks. The *P. gingivalis* + SB group underwent the same operation, except that they received an intraperitoneal injection of SB203580 (5 mg/kg, i.p.) 1 h preoperatively. Three days after the last medication, we conducted a behavioral test in the rats to evaluate their cognitive function. The schematic diagram of experimental process is presented in Figure 1.

As described in a previous study, *P. gingivalis* (strain W83) grew anaerobically (Ilievski et al., 2016). We purchased SB203580 (HY-10256A, NJ, USA) from MedChemExpress.

### The Morris water maze test

Ten weeks following the injection, we tested their spatial learning and memory abilities using the MWM. Water was contained in a round pool (R = 60 cm, H = 50 cm) in the MWM device. The pool was divided into four quadrants: southwest (SW), northwest (NW), northeast (NE), and southeast (SE). There is a platform in the NW quadrant, submerged 2.5 cm under the surface of the water. The task includes a training phase and a testing phase. During the first four consecutive days, the rats were placed in four different quadrants in turn to discover the platform for 60 s. The escape latency was registered when they found the platform within 60 s and stood for more than 5 s. If the platform was not found within 60 s, they were guided to find the platform and stand for 60 s, and the escape latency was recorded as 60 s. On the last day, the rats’ memory ability was tested, the platform was removed, the rats were placed in the pool for 60 s to navigate, and the device recorded relevant data.

### Micro-CT

After the MWM test, we removed ligatures, and the rats were killed by euthanized. A micro-CT scan (Viva CT40; SCANCO Medical, Bruttisellen, Switzerland) was performed on the specimens at a resolution of 15 mm after fixing them in 4% paraformaldehyde. For the volumetric analysis, we used Mimics Analysis software to assess the trabecular thickness (Tb. Th) parameters and bone volume/tissue volume (BV/TV).

### Transcriptome sequencing

Hippocampal tissue samples from different groups of rats were collected, and the transcriptome was sequenced. Total RNA from the hippocampus was extracted from rats using TRIzol (Invitrogen). The hippocampus was ground into powder with liquid nitrogen, and an appropriate volume of TRIzol was added and homogenized for 2 min. Then, the contents were left to stand for 5 min and centrifuged at 12,000 × *g* for 5 min at 4°C. Next, the supernatant was transferred to a new EP tube, and an appropriate amount of chloroform was added. The mixture was shaken for 15 s and allowed to stand for 2 min at room temperature. The tube was centrifuged at 12,000 *g* and 4°C for 15 min. As soon as the upper aqueous phase containing RNA was centrifuged, it was transferred to a new EP tube, isopropanol was added, and the tube was incubated at room temperature for 10 min. The contents were centrifuged at 12,000 × *g* for 10 min at 4°C, followed by decantation of the supernatant and retention of the pellet. Once the pellet was washed with 75% ethanol, it was centrifuged again at 12,000 × *g* for 5 min at 4°C. The supernatant was poured into test tubes and air dried at room temperature for 5 min. The quality of these samples was verified, and mRNA libraries were constructed using an Illumina NovaSeq 6000. This process was performed by Majorbio Biopharm Technology Company, Shanghai, China.

### RT–PCR

Total mRNA from the rat hippocampus was extracted using RNAiso Plus (Takara, Shiga, Japan). The purity and concentration of RNA were measured using 260 and 280 nm ratios of 1.8–2.1. We used the HighCapacity Reverse Transcriptase Kit (Takara) to reverse-transcribe 50 ng of the extracted mRNA. Subsequently, a PCR sample of 10 μl was prepared with 1 μl of cDNA, 5 μl of TB Green Premix EX Taq II (Takara), and 3.2 μl of ddH_2_O. The forward and reverse primers were as follows:

IL-1β: forward: 5′-AACCTGCTGGTGTGTGACGTTC-3′,

Reverse: 5′-CAGCACGAGGCTTTTTTGTTGT-3′;

IL-6: Forward: 5′-GCCCTTCAGGAACAGCTATGA-3′;

Reverse: 5′-TGTCAACAACATCAGTCCCAAGA-3′;

IL-8: Forward: 5′-CATTAATATTTAACGATGTGGATGCG-3′,

Reverse: 5′-GCCTACCATCTTTAAACTGCACAAT-3′;

TNF-α: Forward: 5′-TCAGCCTCTTCTCATTCCTGC-3′,

Reverse: 5′-CGCTTGGTGGTTTGCTACGA-3′;

CRP: Forward: 5′-GTTGGTAGGGTATTCAGCCCC-3′,

Reverse: 5′-TAAGGAGGGACAAGGGAGAGA-3′;

GAPDH: Forward: 5′-ACAGTCCATGCCATCACTGCC-3′,

Reverse: 5′-GCCTGCTTCACCACCTTCTTG-3′.

The data were calculated using the 2^–ΔΔCT^ method.

### ELISA

Approximately 2 ml of blood was collected from the rats and placed in enzyme-free tubes for the assessment of serum cytokines. After centrifugation (4°C, 2,500 rpm, 20 min), the blood supernatant medium was separated for use. For tissue, homogenous samples of hippocampal and gingival samples were prepared using 1% protease inhibitor cocktail (Sigma, St. Louis, MO, USA), radioimmunoprecipitation assay (RIPA) lysis buffer (Beyotime, Beijing, China) and 1% phenylmethylsulfonyl fluoride (PMSF, Beyotime, Beijing, China). The protein levels of TNF-α, IL-1β, IL-6, IL-8, and CRP were assessed using ELISA kits based on the manufacturer’s instructions.

### Paraffin-fluorescence *in situ* hybridization (FISH) and immunofluorescence

Fluorescence *in situ* hybridization is a technique capable of simultaneously visualizing, identifying, enumerating, and localizing microorganisms. These probes specifically hybridize to their complementary target sequences by incorporating short fluorescently labeled probes. Intact cells can be identified at the genus or species level. The procedure consists of five steps: fix microbial samples; pretreat and infiltrate samples to allow access to nucleic acid probes; label probes to hybridize to DNA or RNA targets; wash to remove unbound probes; and mount and visualize by microscopy or flow cytometry (Moter and Göbel, 2000; Prudent and Raoult, 2019). For FISH, sections of brain tissue were placed in fixed fluid (DEPC) 12 h after cleaning, embedded in paraffin and sectioned. The hybridization protocol was conducted as previously reported (Sakuraoka et al., 2012). The probe for the FISH analysis was POGI: 5′-CAATACTCGTATCGCCCGTTATTC-3′. The sections were placed in the probe hybridization solution in a constant temperature box and hybridized overnight, and the sections were then washed with PBS.

For the immunofluorescence assay, sections of brain tissue were first incubated with 3% H_2_O_2_ in methanol, blocked with 10% goat serum, incubated overnight at 4°C with ionic calbindin adaptor molecule (Iba1) (1:400, ARG63338; Arigo, Biolaboratories, Hsinchu, Taiwan, China) and then counterstained with DAPI with a secondary antibody (Abcam; 1:3,000) at 37°C for 1 h.

### Western blot

The rat hippocampal tissue was homogeneously dissolved in protease inhibitor and protein lysis buffer, and the protein concentration was determined with a BCA protein analysis kit. Equal amounts of protein were heated at 100°C for 5 min, after which they were separated using 10% SDS polyacrylamide gel electrophoresis and transferred to PVDF membranes (0.45 μm) blocked with 5% skim milk. Proteins were detected with the corresponding antibodies, including anti-amyloid precursor (APP, 1:5,000, ab32136, Abcam, Cambridge, UK), BACE1 (1:1,000, no. 5606; Cell Signaling Technology, United States), P38 MAPK (1:1,000, ab31828, Abcam, Cambridge, UK), P-P38 (1:1,000, ab47363, Abcam, Cambridge, UK), and β-actin (1:5,000, ab32572, Abcam, Cambridge, UK).

### Statistical analysis

The data are expressed as the mean ± standard error of the mean (SEM). GraphPad Prism software and SPSS software version 26.0 were used for one-way and two-way ANOVA to calculate the p value (except that the latency in the MWM test was evaluated using two-way ANOVA; the other parameters were assessed used one-way ANOVA). There was statistical significance in the ANOVA using Tukey’s *post-hoc* multiple comparison test at *p* < 0.05.

## Results

### Assessment of the extent of alveolar bone resorption

**FIGURE 2:**
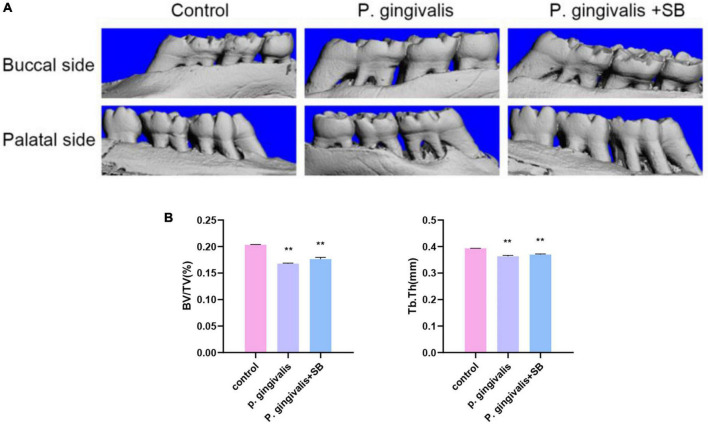
Effects of topical application of *P. gingivalis* into the periodontal pocket of maxillary first molars on alveolar bone. **(A)** Images captured by micro-CT scanning of the molar region. Bone density analysis of alveolar bone including **(B)** BV/TV, Tb.Th. Experimental values are expressed as the mean ± SEM (*n* = 3 per group). **p < 0.01 compared with the control group.

As demonstrated in Figure 2A, there was buccal and palatal alveolar bone resorption in the *P. gingivalis* and *P. gingivalis* + SB groups, and the resorption was visually more severe in the *P. gingivalis* group which was compared with the control group. Meanwhile, we assessed BV/TV and Tb. Th (Figure 2B) and found that the absorption in the *P. gingivalis* group and *P. gingivalis* + SB group were more severe.

### Effects of periodontitis on spatial learning and memory ability

**FIGURE 3:**
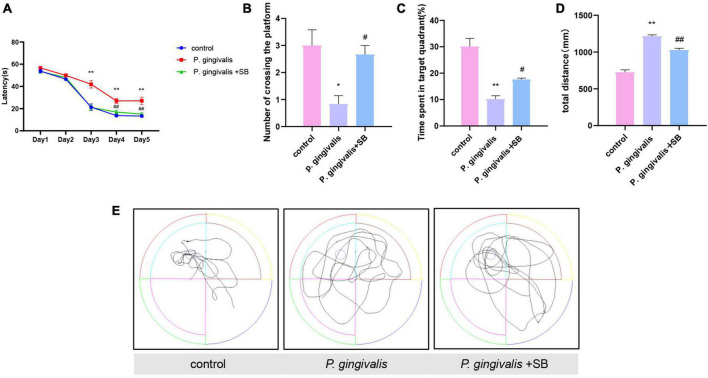
Effects of periodontal inflammation on spatial learning and memory in the MWM test. **(A)** Latency in finding the platform during the acquisition phase of the MWM test. **(B)** Number of platform crossings in the target quadrant. **(C)** Percentage of time spent in the target quadrant. **(D)** The total distance traveled by rats in the MWM test. **(E)** The typical trajectory. The platform is represented by a circle. Experimental values are expressed as the mean ± SEM (*n* = 8 per group). *p < 0.05 and **p < 0.01 compared with the control group, ^#^*p* < 0.05 and ^##^*p* < 0.01 compared with the *P. gingivalis* group.

The MWM test was used to evaluate whether periodontitis could contribute to the learning and memory ability of rats. As shown in Figure 3A, the escape latency of rats in each group gradually decreased in the first 5 days of training. The escape latency of the *P. gingivalis* group was significantly longer than that of the control group, and there was no significant difference between the control group and the *P. gingivalis* + SB group. On the 6th day, we removed the platform, and the number of times the rats crossed the platform and the percentage of time spent in the target quadrant were assessed. The results showed that the *P. gingivalis* group had fewer platform crossings and a shorter percentage of time spent in the target quadrant than the control group (Figures 3B, C). Meanwhile, the rats in the *P. gingivalis* group moved a greater total distance (Figure 3D). As seen from the animal trajectories, rats in the control group could navigate directly to the target representation where the platform was located; however, this behavioral ability was impaired in the *P. gingivalis* group (Figure 3E). This change could be reversed by SB203580. These results suggested that *P. gingivalis*-induced periodontitis can be seen as a risk factor for cognitive impairment.

### Results of transcriptome sequencing of hippocampal tissue

**FIGURE 4:**
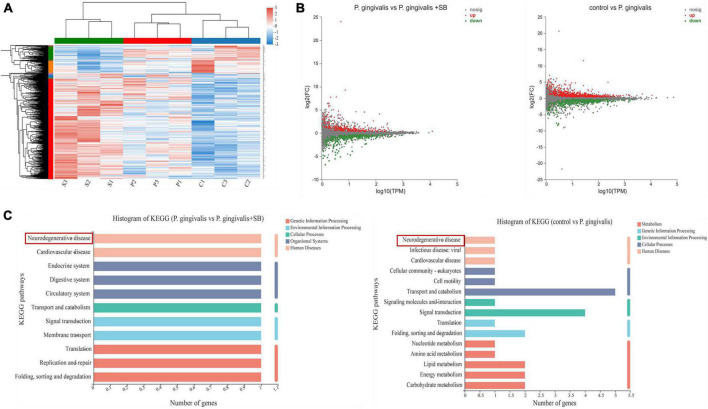
The transcriptome sequencing results. **(A)** Heatmaps of significantly changed genes after treatment (|log| ≥ 2, *p* < 0.05) (*n* = 3 per group. “s” refers to the *P. gingivalis* + SB group, “p” refers to the *P. gingivalis* group, and “c” refers to the control group). **(B)** MA plot of the difference in expression between *P. gingivalis* and the control group and vs. *P. gingivalis* + SB. **(C)** The 20 most significantly enriched KEGG metabolic pathways.

We used transcriptomics analyses to further elucidate the underlying mechanisms of these module rats. Heatmaps of the DEGs after treatment are shown in Figure 4A. This result indicates that our experimental animals are reproducible. It was found in the MA plot (Figure 4B) that the *P. gingivalis* group exhibited differences in the expression of 960 genes, 614 and 346 of which were upregulated and downregulated, respectively, compared with that in the control group. Compared with the *P. gingivalis* group, 554 genes were differentially expressed, 267 and 287 of which were upregulated and downregulated, respectively. After mining the differential gene data, KEGG functional annotation analysis was performed on the differential genes. The results are shown in Figure 4C, which displays the top 20 KEGG pathways with the greatest abundance. After comparing the control group with the *P. gingivalis* group, it was found that neurodegenerative diseases were associated with the gene pathways with differential expression. Comparing the *P. gingivalis* group and *P. gingivalis* + SB group, neurodegenerative diseases were also associated with differentially expressed gene pathways. This result means that we successfully modeled neurodegeneration. In addition, we found that signaling pathways are also present in DEGs.

### The expression levels of inflammatory factors in gingival tissue

**FIGURE 5:**
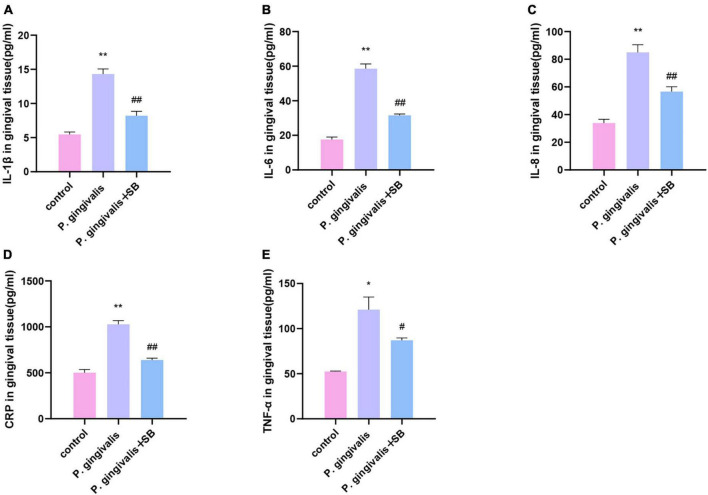
Effects of periodontal inflammation in the gingival tissue. ELISAs were performed to detect the protein levels of inflammatory cytokines. **(A)** IL-1β; **(B)** IL-6; **(C)** IL-8; **(D)** CRP; **(E)** TNF-α. Experimental values are expressed as the mean ± SEM (*n* = 4 per group). *p < 0.05 and **p < 0.01 compared with the control group, ^#^*p* < 0.05 and ^##^*p* < 0.01 compared with the *P. gingivalis* group.

The protein levels of TNF-α, IL-1β, IL-6, IL-8, and CRP in the gingival tissue in the *P. gingivalis* group were significantly higher than those in the control group, and the levels of these inflammatory factors in the *P. gingivalis* + SB group were lower than those in the *P. gingivalis* group (Figures 5A–E).

### The level of inflammatory factors in serum

**FIGURE 6:**
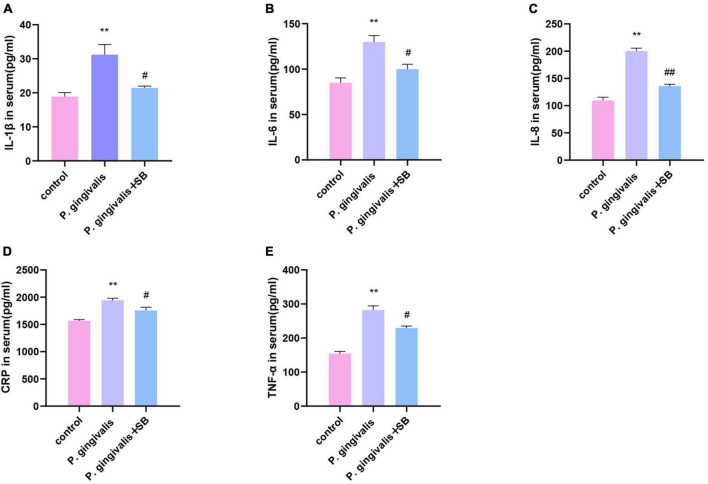
Effects of periodontal inflammation in the serum. ELISAs were performed to detect the protein levels of inflammatory cytokines. **(A)** IL-1β; **(B)** IL-6; **(C)** IL-8; **(D)** CRP; **(E)** TNF-α. Experimental values are expressed as the mean ± SEM (*n* = 4 per group). **p < 0.01 compared with the control group, ^#^*p* < 0.05 and ^##^*p* < 0.01 compared with the *P. gingivalis* group.

ELISA was performed to determine the protein levels of TNF-α, IL-1β, IL-6, IL-8, and CRP in serum (Figure 6). The levels in the *P. gingivalis* group were significantly higher than those in the control group. Compared with the *P. gingivalis* group, the expression of these factors in the *P. gingivalis* + SB group was decreased. This result shows that SB203580 could reverse this change.

### Effects of inflammatory factors induced by periodontal inflammation in the hippocampus

**FIGURE 7:**
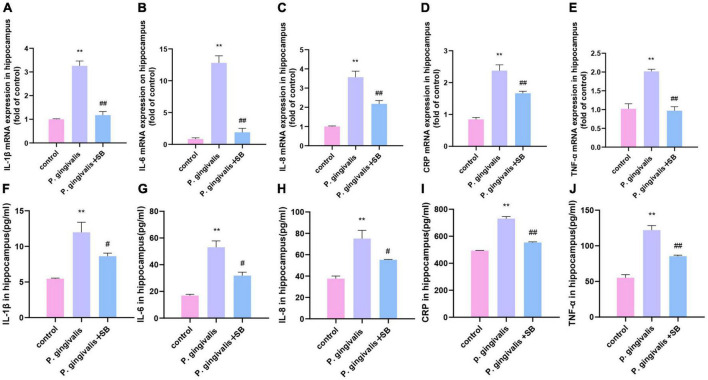
Effects of periodontal inflammation in the hippocampus. q-PCR and ELISA were performed to detect the mRNA and protein levels of inflammatory cytokines in the hippocampus. The mRNA levels of cytokines in the hippocampus in each group: **(A)** IL-1β; **(B)** IL-6; **(C)** IL-8; **(D)** CRP; and **(E)** TNF-α. The protein levels of cytokines in the hippocampus in each group: **(F)** IL-1β; **(G)** IL-6; **(H)** IL-8; **(I)** CRP; and **(J)** TNF-α. Experimental values are expressed as the mean ± SEM (*n* = 4 per group). **p < 0.01 compared with the control group, ^#^*p* < 0.05 and ^##^*p* < 0.01 compared with the *P. gingivalis* group.

We further detected mRNA and protein levels in the hippocampus. Compared with the control group, the *P. gingivalis* group had higher mRNA and protein levels of TNF-α, IL-1β, IL-6, IL-8, and CRP. Compared with the *P. gingivalis* group, the *P. gingivalis* + SB group had decreased levels of these factors (Figures 7A–J). These results suggest that *P. gingivalis*-induced periodontitis can increase the level of inflammatory factors in the brain, and the change can be prevented to a certain extent by SB203580.

### *P. gingivalis* can be found in the hippocampus

**FIGURE 8:**
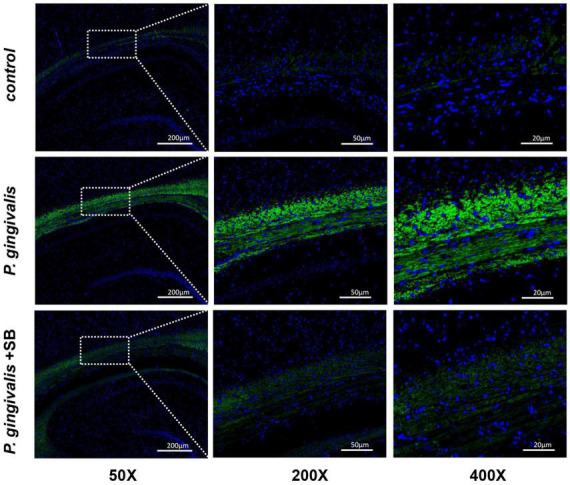
Expression of *P. gingivalis* in the hippocampal tissue as assessed by FISH. Blue represents the nucleus, and green is the region where *P. gingivalis* is present (*n* = 3 per group) (50×, 200×, and 400×, bar = 200 μm, bar = 50 μm, and bar = 20 μm).

As shown in Figure 8, the *P. gingivalis* group had more *Porphyromonas gingivalis* colonization, while the control group had the least amount. This result indicates that the content of *P. gingivalis* in the brain has a certain positive relationship with cognitive impairment.

### Effects of inflammation on microglia in the hip

**FIGURE 9:**
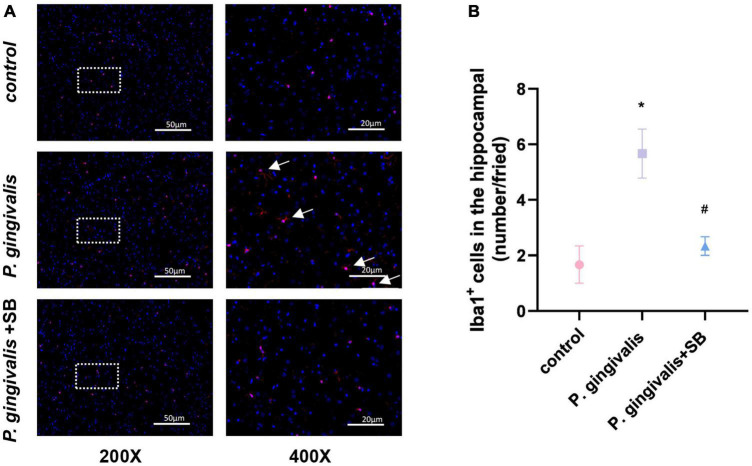
Effects of periodontitis on microglia in the hippocampus. Histopathological analysis of brain sections was performed using immunofluorescence. **(A)** Microglia were visualized with Iba1 (red). White arrows indicate activated microglia. Quantification of Iba1 levels in the hippocampus is shown. **(B)** Iba1-positive cells in the hippocampus (number/field). Experimental values are expressed as the mean ± SEM (*n* = 3 per group) (200× and 400×, bar = 50 and 20 μm). *p < 0.05 compared with the control group, ^#^*p* < 0.05 compared with the *P. gingivalis* group.

Immunofluorescence results showed activation of microglia in the hippocampus of the *P. gingivalis* group. We observed an increase in microglial endpoints and irregular protrusions, which were ameliorated by treatment with SB203580 (Figures 9A, B). This phenomenon indicates that inflammation plays a role in the brain.

### Effects of periodontitis on APP processing and the BACE1 and P38 MAPK pathways

**FIGURE 10:**
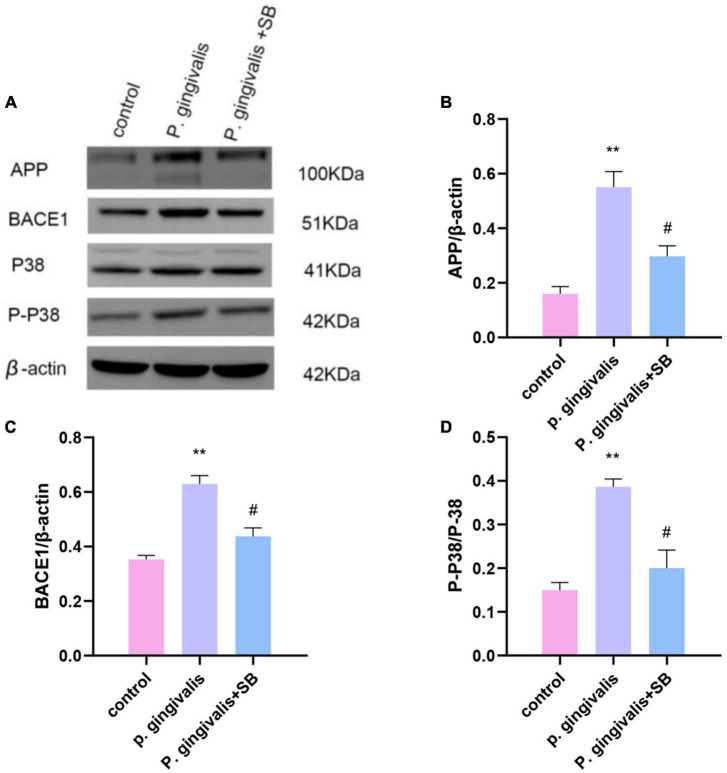
Effects of periodontitis on the protein level of APP and the activation of the P38 MAPK signaling pathway. **(A)** All of the above proteins were upregulated by *P. gingivalis* (except protein P38). High expression of these proteins was effectively inhibited by SB203580. **(B–D)** The quantification of related protein expression in each group. Experimental values are expressed as the mean ± SEM (*n* = 3 per group). **p < 0.01 compared with the control group, ^#^*p* < 0.05 compared with the *P. gingivalis* group.

Compared with the control group, the *P. gingivalis* group had higher protein expression of APP and BACE1. Moreover, we found that the *P. gingivalis* group had increased protein levels of P-P38/P38 MAPK, which were reduced by SB203580 (Figures 10A–D). This result suggests that periodontitis may trigger neurodegeneration through the P38 MAPK signaling pathway.

## Discussion

In this study, we demonstrated that silk thread ligature-induced periodontitis plus injection of *P. gingivalis* into subgingival tissue could induce memory and cognitive impairment. Transcriptome sequencing results suggested that there were neurodegenerative diseases in the *P. gingivalis* group, and the MWM test demonstrated that periodontitis reduced the cognitive ability of MCI model rats. We found high expression of inflammatory factors (TNF-α, IL-1β, IL-6, and IL-8) and CRP in the gingiva, peripheral blood and hippocampus, and the expression of APP and BACE1 and activation of the P38 MAPK pathway were increased. Activated microglia and the presence of *P. gingivalis* were also found in the hippocampus. P38 MAPK inhibition could alleviate all the above changes.

Periodontitis is considered a possible risk factor for cognitive impairment. Researchers have performed many studies to explore their association. Zhang et al. (2018), Liu et al. (2020) used intraperitoneal injection of *P. gingivalis*-lipopolysaccharide to simulate the periodontal-induced Chronic Low-Grade Inflammatory Phenotype (CLIP) to study its relationship with MCI. Ilievski et al. (2018) used oral application of *P. gingivalis* to explore their relationship. Their final results confirmed that chronic periodontal pathogen infection can lead to the development of neuropathology consistent with neurodegenerative disease. Since the above scholars have not proven the existence of inflammation in the periodontal tissue, whether by intraperitoneal injection or oral local application, they have not directly constructed a periodontitis model to study their relationship. Therefore, some scholars have improved the experimental method. Some researchers have constructed this model by simply ligating the molars with wire or injecting *P. gingivalis*-lipopolysaccharide into the gingiva. Their experimental results also demonstrated that periodontitis can lead to a diminution in spatial cognitive ability in rats, eventually contributing to neuroinflammation and cognitive impairment (Hu et al., 2020, 2021).

*Porphyromonas gingivalis* is the main pathogenic bacterium of periodontitis, and its pathogenicity is attributed to a series of virulence factors, such as lipopolysaccharides, capsula polysaccharides, etc. The lipopolysaccharides of *P. gingivalis* are composed of lipid A, core oligosaccharides and O-Antigenic Regions, which have a strong pathogenic effect in periodontal tissues (Fitzsimmons et al., 2018). In addition, the capsular polysaccharides of *P. gingivalis* as special mucous polymers on the surface of bacteria, are the main inducers of immune responses. As one of the virulence factors, it can inhibit the host’s immune defense response and play an immune escape role to achieve the goal of persistent infection. At present, there are at least six serotypes of this bacterium, namely K1-K6 serotype. Vernal et al. (2009) have reported that capsular serotype K1 and K2 *P. gingivalis* induce an enhanced secretion of pro-inflammatory interleukin cytokines, implying capsular *P. gingivalis* have the armory for potentiating BBB permeability through cytokine liberation. *P. gingivalis* W83, as a representative strain of K1 serotype, can enhance the colonization ability of bacteria through lipopolysaccharide or capsular polysaccharide on its surface, make bacteria more toxic, and increase the risk of neurodegenerative diseases (Kanagasingam et al., 2020).

To better simulate the characteristics of clinical periodontitis and to study how *P. gingivalis*, as the main pathogen of periodontitis, induces neuroinflammation through periodontitis, we constructed this model by ligating the first molar with silk thread to create a local periodontal pocket and injected *P. gingivalis* into the periodontal pocket. Silk thread ligation, compared with wire ligation, as a classic method of constructing periodontitis, minimizes the mechanical damage to the periodontal tissue. From many previous studies, it has been concluded that subgingival plaque can enter the systemic circulation through broken periodontal pockets, triggering CLIP (Kuraji et al., 2021). We used silk ligation to promote the accumulation of subgingival plaque. This approach can promote the formation of periodontal pockets and provide a persistent inflammatory microenvironment that promotes the continued progression of periodontitis, which in turn triggers CLIP. Our experimental results showed obvious resorption of the alveolar bone, indicating that we successfully constructed a periodontitis model (Figures 2A, B). The results of behavioral experiments showed that there were differences between the experimental and control groups, which indicated that the rats had cognitive impairment (Figures 3A–E). Transcriptome sequencing also confirmed this result. When we mined the differential genes in each group, we found that there were neurodegenerative disease-related genes in the *P. gingivalis* group (Figures 4A–C).

Inflammation plays a significant role in the destruction of periodontal tissue induced by periodontitis. The immediate response to periodontal pathogens and their endotoxins is to activate the host immune response, releasing inflammatory cytokines into systemic circulation (Sureda et al., 2020). Periodontal disease elicits host immune responses, including innate and acquired immune responses. At this time, inflammatory cells (macrophages, plasma cells, T and B lymphocytes) infiltrate the periodontal tissue and release inflammatory factors. In the regulation of anti-inflammatory and proinflammatory responses, the inflammatory response expands and activates the MAPK signaling pathway to gradually trigger CLIP (Ohlrich et al., 2009). The outcomes of our ELISA showed that the inflammatory factors in the periodontal tissue of the first molars ligated in the experimental group increased (Figures 5A–E). Simultaneously, the ELISA results of the serum of the experimental group showed that these inflammatory factors were also increased compared with those in the control group (Figures 6A–E). This phenomenon indicates that local periodontal inflammatory factors may enter the bloodstream and cause CLIP. CLIP may damage organs such as distant brain tissue (Snowdon et al., 1997). Periodontitis is considered a possible risk factor for cognitive impairment because of the systemic inflammatory burden from infection.

Periodontitis can cause neuroinflammation in the brain in the following ways: periodontal pathogens or their virulence factors invade the brain tissue directly via the blood or cranial nerves. As mentioned earlier, *P. gingivalis* is described as a key pathogen in periodontitis (Olsen et al., 2016). Poole et al. (2013) have shown that *P. gingivalis* components are identified in cognitive impairment subjects, which could help to explain the association of periodontitis with the beginning of perpetuating inflammation. A recent study validated the presence of *P. gingivalis* in the brain using ApoE mice to establish periodontitis, where mice were topically administered *P. gingivalis*, and *P. gingivalis* genomic DNA was found in mouse brains (Poole et al., 2015). In addition, Díaz-Zúñiga et al. (2020) detected the presence of the bacteria in serum, cerebrospinal fluid (CSF), and hippocampus by quantifying the 16S rRNA subunit of *P. gingivalis* by qPCR after inoculation with *P. gingivalis* in the palatine mucosa. These results suggest that *P. gingivalis* can enter the brain. Similarly, our FISH results showed that the content of *P. gingivalis* in the *P. gingivalis* group was higher than that in the control group (Figure 8). The indirect mechanism is that periodontal pathogens and their virulence factors can activate microglia by stimulating the pia mater, causing an inflammatory response in the brain and triggering neuroinflammation (D’Mello and Swain, 2017; Hashioka et al., 2019). We observed increased levels of inflammatory factors in the hippocampus and more activated microglia in the immunofluorescence results in our experimental group (Figures 7, 9). During chronic periodontitis, driven by the active ingredients of periodontal pathogens, the production of IL-1β and TNF-α stimulates microglia in the brain, and they act as the key causative agents of MCI and can stimulate the release of more inflammatory factors by activating microglia, further promoting the development of MCI (Schwab and McGeer, 2008). IL-6 is probably the most important cytokine involved in microglial activity and inflammatory responses. It plays an important role in neuronal degeneration, causing neuronal degeneration and cell death (Gadient and Otten, 1997). IL-8, as an inflammatory factor, also triggers neurological diseases (Ellman et al., 2010). Elevation of CRP, an acute-phase reactive protein, increases the risk of cognitive impairment (Engelhart et al., 2004). From our experimental results, it can be seen that these inflammatory factors are found in gingival tissue, peripheral blood and hippocampal tissue (Figures 5–7). This phenomenon demonstrates another mechanism through which periodontitis triggers MCI; specifically, peripheral proinflammatory cytokines associated with periodontitis can cause neuroinflammation in the brain through a peripheral pathway. Thus, from all the above results, we reasonably speculate that periodontitis triggers MCI by triggering neuroinflammation.

It is generally believed that MCI is triggered by neuroinflammation. p38 MAPK contributes to neuroinflammation mediated by microglia (Cargnello and Roux, 2011). Once there is an inflammatory process in the brain, neurodegeneration, synaptic dysfunction, and cognitive dysfunction may occur (Kamer et al., 2008). Under the action of inflammation, microglia are activated. Activated microglial cells produce a range of inflammatory factors (Perry et al., 2010; Wu and Nakanishi, 2014). These inflammatory mediators can upregulate APP levels and BACE1 activity in the brain via P38 MAPK signaling. The accumulation of these neuropathological markers will further lead to a decline in the body’s learning and memory functions (Mantzavinos and Alexiou, 2017; Kheiri et al., 2018). Our experimental results showed that in addition to the increased inflammatory factors seen in the hippocampus of the experimental group, increased protein expression of APP and BACE1 was also observed, which was reversed by SB203580 (Figures 10A–D).

Numerous studies and our experimental results have confirmed that local periodontal inflammation may induce inflammatory responses throughout the whole body and brain tissue, resulting in changes in the morphology of microglia, but the specific mechanisms of periodontal inflammation and MCI are still not well clarified. Hu et al. (2021) showed that periodontitis in rats induced by ligation leads to systemic inflammation and aberrant APP processing, leading to cognitive impairment. In this progression, the activation of the STAT3 pathway may play a crucial role by raising the inflammatory burden and promoting neuroinflammation. Hu et al. (2020) noted that periodontitis caused by topical application of *P. gingivalis*-LPS may lead to impaired learning and memory ability in SD rats through activation of TLR4/NF-κB pathway-induced neuroinflammation. This process may involve abnormal APP processing. These results suggest that periodontitis can not only cause damage to periodontal support tissue but also activate certain pathways (STAT/TLR/NF-κB) that trigger systemic inflammation throughout the body, ultimately leading to cognitive impairment. MAPK pathways represent ubiquitous signal transduction pathways which is expressed in the mature central nervous system in response to various extern stimuli (Origlia et al., 2009). MAPK signaling can promote neuropathic inflammation, amyloid-beta toxicity and apoptosis (Kheiri et al., 2018). Some researchers have suggested that MAPKs are upstream signaling mediators of many inflammatory factors, such as TNF-α and IL-1β. Moreover, cytokines can be directly or indirectly regulated by P38 MAPK (Chi et al., 2006; Cook et al., 2007), which plays a significant role in the proinflammatory signaling network (Lee and Kim, 2017). Indeed, P38 MAPK plays a critical role in normal immune and inflammatory responses, various studies have revealed its involvement in the production of inflammatory cytokines leading to chronic inflammation (Lee and Kim, 2017). Many studies have revealed P38 MAPK is classically known as a responsive element to neuroinflammation which is crucial for learning and memory and the P38 MAPK cascade is activated in the presence of specific pathological markers (Lee et al., 2000; Falcicchia et al., 2020). P38 inhibitors have potential beneficial outcomes in LPS-induced periodontitis and neurodegenerative diseases (Kirkwood and Rossa, 2009; Mansour et al., 2021). Our study suggested that P38 MAPK activation by *P. gingivalis*, leaded to periodontitis, may play a crucial role in the formation and development of neuroinflammation in cognitive impairment. Additionally, it was found that use of the P38 inhibitor SB203580 resulted in opposite results to those found in the *P. gingivalis* group.

There are still some deficiencies in this experiment, which are mainly reflected in the following: first, although we carefully insert the microsyringe into the bottom of the subgingival pocket on the palatine side to inject the suspension of *P. gingivalis*. Still, there will also be minimal bacterial fluid spillage, which is an unavoidable problem during the experiment; this experiment mainly studied the role of pro-inflammatory factors in the process of periodontitis causing MCI, and did not fully consider anti-inflammatory factors such as IL-10; finally, in the *in situ* hybridization results of the *P. gingivalis* + SB group, it can be seen that the amount of bacteria in brain tissue is less than that in the *P. gingivalis* group, and the specific mechanism of this result needs to be further studied.

## Conclusion

Our results strongly suggest that ligating plus injecting *P. gingivalis* into subgingival tissue can increase the inflammatory response in both the periphery and CNS, contributing to cognitive impairment via neuroinflammation, which is induced by P38 MAPK signaling pathway activation in SD rats. It can also modulate APP processing. Therefore, P38 MAPK may serve as a linking pathway between periodontitis and cognitive impairment.

## Data availability statement

The RNA sequencing data presented in the study are publicly available. This data can be found here: NCBI repository, accession number PRJNA924699. The original contributions presented in this study are included in the article/Supplementary material, further inquiries can be directed to the corresponding author.

## Ethics statement

This project has been approved by the Ethics Committee of the Stomatological Hospital of Chongqing Medical University, Chongqing, China.

## Author contributions

RJ conceived and performed experiments, analyzed data, and wrote and edited the manuscript. XN performed some experiments and formal analysis. XL and YZ performed some experiments. GY provided financial and technical support, analyzed data, and wrote the manuscript. All authors contributed to the article and approved the submitted version.

## Supplementary material

Supplementary Material Part of uncropped blots.
