# Supplementary figures and images for: Porphyromonas gingivalis-induced periodontitis could contribute to cognitive impairment in Sprague–Dawley rats via the P38 MAPK signaling pathway
Source: Front Cell Neurosci. 2023 Mar 28;17:1141339. doi: 10.3389/fncel.2023.1141339 (PMC10086325; doi:10.3389/fncel.2023.1141339)

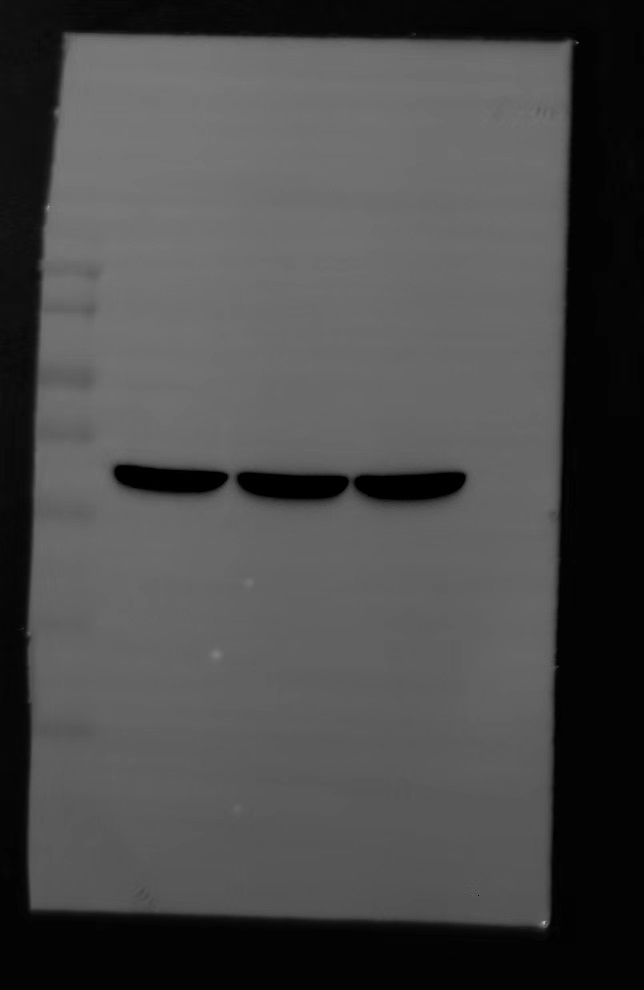

Supplement: Supplementary file 1 [file Image_1.JPEG]

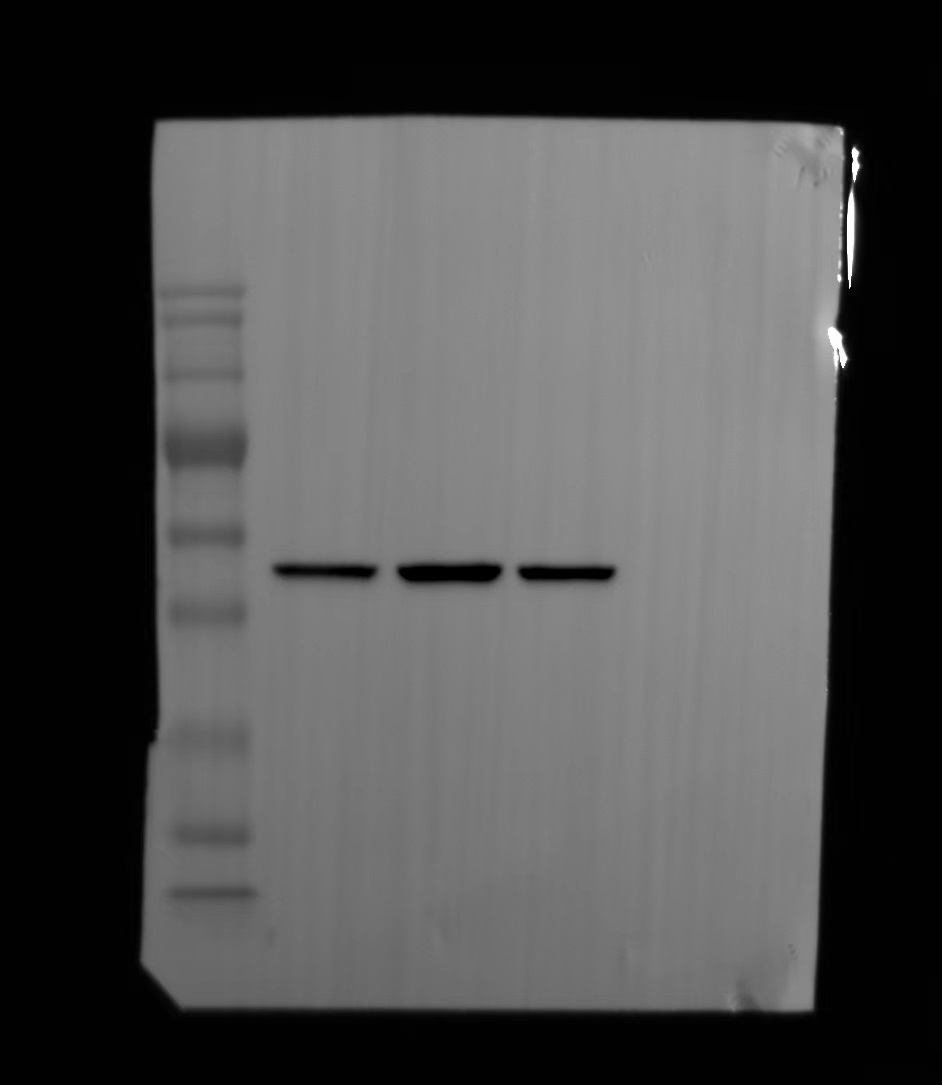

Supplement: Supplementary file 2 [file Image_2.JPEG]

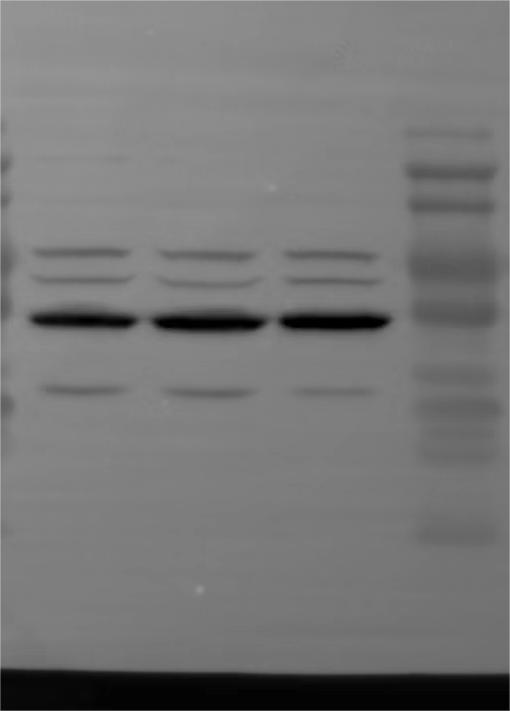

Supplement: Supplementary file 3 [file Image_3.JPEG]

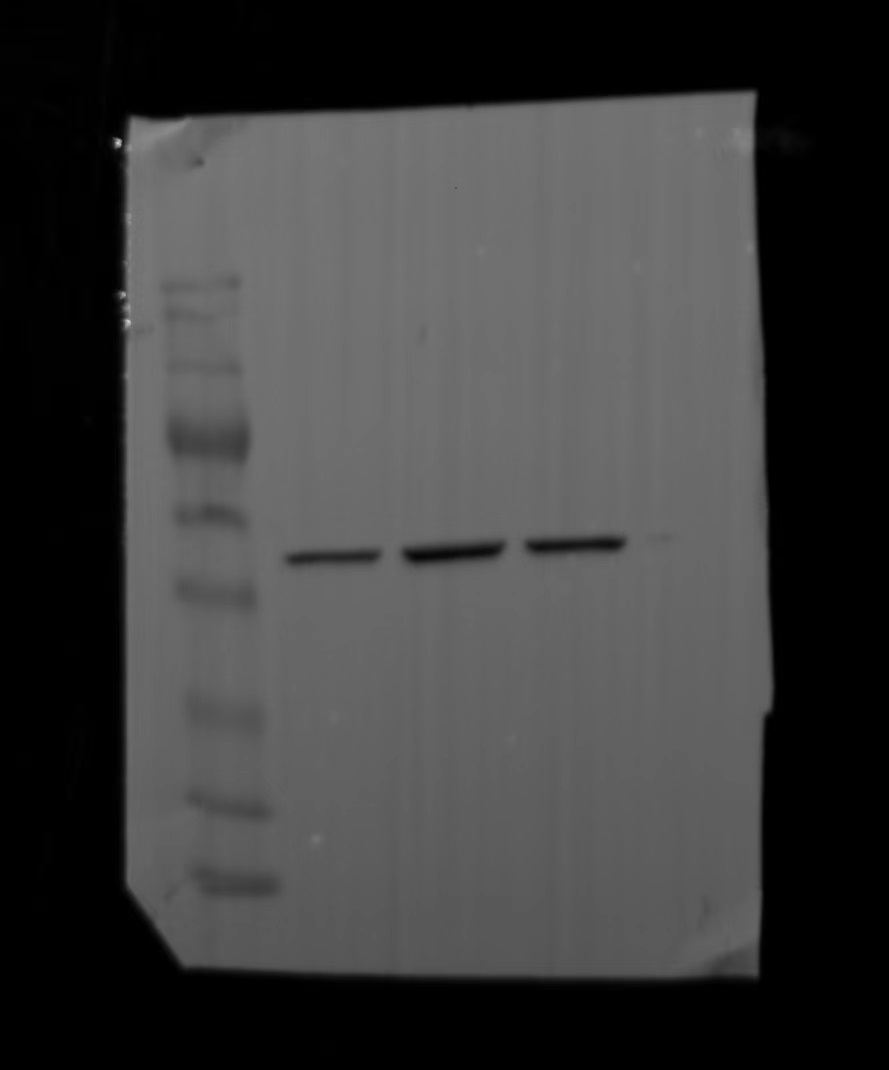

Supplement: Supplementary file 4 [file Image_4.JPEG]

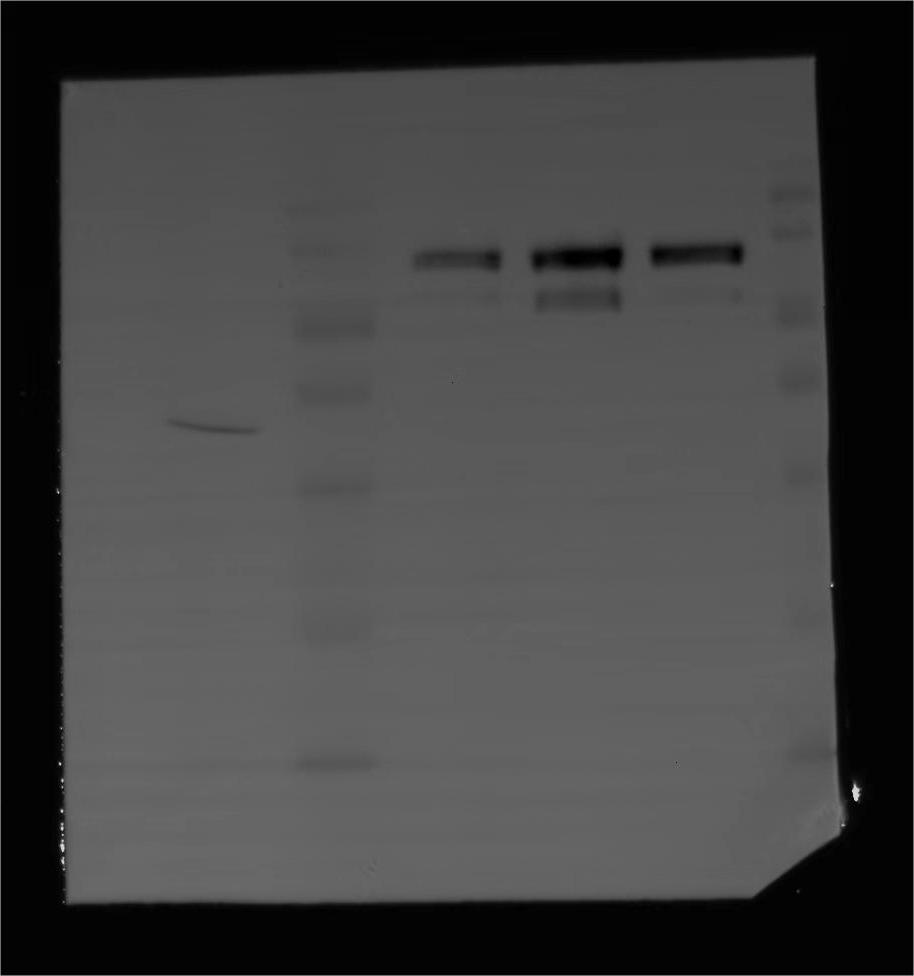

Supplement: Supplementary Material — Part of uncropped blots. [file Image_5.JPEG]
